# A Comparative Analysis of MicroRNA Expression in Mild, Moderate, and Severe COVID-19: Insights from Urine, Serum, and Nasopharyngeal Samples

**DOI:** 10.3390/biom13121681

**Published:** 2023-11-21

**Authors:** Raya Soltane, Nuha Almulla, Ahlam Alasiri, Nabila F. Elashmawy, Alaa T. Qumsani, Fatimah M. Alshehrei, Doaa El-Ghareeb Keshek, Taha Alqadi, Saleh Bakheet AL-Ghamdi, Abdou Kamal Allayeh

**Affiliations:** 1 Department of Biology, Adham University College, Umm Al-Qura University, Makkah 21955, Saudi Arabia; namull@uqu.edu.sa (N.A.); ajasiri@uqu.edu.sa (A.A.); taqadi@uqu.edu.sa (T.A.); 2 Biology Department, College of Science, Jazan University, Jazan 82817, Saudi Arabia; nelashmawy@jazanu.edu.sa; 3 Department of Biology, Jumum College University, Umm Al-Qura University, P.O Box 7388, Makkah 21955, Saudi Arabia; atqumsani@uqu.edu.sa (A.T.Q.); fmshehrei@uqu.edu.sa (F.M.A.); dekeshek@uqu.edu.sa (D.E.-G.K.); 4 Agriculture Genetic Engineering Research Institute (AGERI), Agriculture Research Centre, Giza 12512, Egypt; 5 Biology Department, Faculty of Science, Al-Baha University, Al Baha 65947, Saudi Arabia; sb.alghamdi@bu.edu.sa; 6 Virology Lab 176, Environment and Climate Change Institute, National Research Centre, Giza 12622, Egypt

**Keywords:** SARS-CoV-2, COVID-19, nasopharyngeal, plasma, urine and MicroRNA expression

## Abstract

COVID-19, caused by the SARS-CoV-2 virus, manifests with a wide range of clinical symptoms that vary from mild respiratory issues to severe respiratory distress. To effectively manage and predict the outcomes of the disease, it is important to understand the molecular mechanisms underlying its severity. This study focuses on analyzing and comparing the expression patterns of microRNAs (miRNAs) in serum, urine, and nasopharyngeal samples from patients with mild, moderate, and severe COVID-19. The aim is to identify potential associations with disease progression and discover suitable markers for diagnosis and prognosis. Our findings indicate the consistent upregulation of miR-21, miR-146a, and miR-155 in urine, serum, and nasopharyngeal samples from patients with mild COVID-19. In moderate cases, there were more significant changes in miRNA expression compared to mild cases. Specifically, miR-let-7 demonstrated upregulation, while miR-146b exhibited downregulation. The most notable alterations in miRNA expression profiles were observed in severe COVID-19 cases, with a significant upregulation of miR-223. Moreover, our analysis using Receiver-operating characteristic (ROC) curves demonstrated that miR-155, miR-let-7, and miR-223 exhibited high sensitivity and specificity, suggesting their potential as biomarkers for distinguishing COVID-19 patients from healthy individuals. Overall, this comparative analysis revealed distinct patterns in miRNA expression. The overlapping expression patterns of miRNAs in urine, serum, and nasopharyngeal samples suggest their potential utility in discriminating disease status.

## 1. Introduction

COVID-19, a new respiratory infection caused by the SARS-CoV-2 virus, was first detected in Wuhan in December 2019 and has led to a significant number of deaths worldwide [1]. The prognosis of SARS-CoV-2 infection varies from mild to severe, including conditions like acute respiratory distress syndrome (ARDS) and mortality. Factors such as age, immune response, and underlying health conditions play a role in determining the severity of the illness [2,3]. The host’s response to the infection has a significant impact on the clinical outcomes observed in individuals. Increased levels of cytokines and chemokines, particularly IL-6, IL-8, and tumor necrosis factor, along with lymphopenia and immune cell infiltration in affected organs, are known to contribute to the severity of COVID-19 and the accompanying hyper-inflammatory responses [4,5,6,7].

Clinical literature classifies COVID-19 patients into mild, moderate, or severe categories, but there is a growing consensus among experts that utilizing blood tests to interpret disease progression may yield more accurate results [8]. Given the limited available data, there is a need to identify new non-invasive biomarkers that can differentiate between different disease stages of COVID-19. This requires a deeper understanding of the interactions between the virus, host cells, viral pathogenesis, and cellular damage [9].

In the ongoing global effort to combat the COVID-19 pandemic, scientists are actively engaged in studying the underlying molecular mechanisms of the disease. One area of particular interest is the role of microRNAs (miRNAs). MicroRNAs are RNA molecules that do not code for proteins but instead regulate gene expression after transcription by inhibiting translation. Numerous studies have highlighted the crucial roles of microRNAs in controlling various biological processes such as inflammation, apoptosis, cell proliferation, and the immune response to viral infections [10,11]. Changes in the levels of cellular miRNAs have been associated with the immune response to respiratory viruses, playing a role in activating antiviral and inflammatory pathways [12]. MiRNAs have emerged as potential indicators to identify various diseases, including viral infections, due to their high sensitivity and specificity [13]. Additionally, during the severe phase of infection, specific human miRNAs exhibit different expression patterns compared to the moderate phase, suggesting their potential as prognostic biomarkers [14].

Recently, miRNAs have emerged as potential key contributors to the development and progression of COVID-19. For example, studies suggest that miR-146 may play a role in regulating immune responses and controlling inflammation. Dysregulation of this specific miRNA has been observed in viral infections, including respiratory viruses. Its potential involvement in COVID-19 is currently being explored, with initial evidence suggesting a potential link to disease severity and immune dysregulation. Similarly, miR-193-5p and miR-323a-3p have been associated with renal function and have been linked to kidney injury. In the context of COVID-19, the virus can cause damage to various organs, including the kidneys. Altered expression of these miRNAs may contribute to the renal complications observed in severe cases of COVID-19 [15,16].

Furthermore, miR-17-5p, miR-142-5p, miR-25-5p, miR-29-3p, miR-19b-3p, miR-23a-3p, miR-92a-3p, and miR-320a are involved in immune regulation and cardiovascular function [17,18]. Other miRNAs, such as miR-21, miR-let-7, miR-146a, miR-146b, miR-155, miR-223, and miR-342, are involved in immune regulation, inflammation, lung injury, and fibrosis. For instance, miR-21 plays a role in immune regulation and inflammation and has been associated with lung injury and fibrosis, indicating its potential contribution to disease progression [19,20]. Similarly, miR-let-7 is a group of miRNAs involved in immune regulation and inflammation, affecting immune cell function and cytokine production. Dysregulation of miR-let-7 has been linked to lung injury and fibrosis, suggesting its potential involvement in these conditions [20].

MiR-146a acts as a crucial regulator of inflammation and immune responses, functioning as a negative feedback regulator by targeting genes involved in pro-inflammatory signaling pathways. Dysregulation of miR-146a has been observed in various inflammatory diseases and has been associated with lung injury and fibrosis due to its impact on inflammatory processes. Similarly, miR-146b is another member of the miR-146 family, which shares similar regulatory functions with miR-146a, acting as a negative regulator of inflammation by targeting key signaling molecules involved in immune responses [21,22].

Moreover, miR-155 is a well-known regulator of immune responses and inflammation. It is expressed in different immune cells and can influence the production of pro-inflammatory cytokines. Dysregulation of miR-155 has been implicated in several inflammatory diseases, and its role in lung injury and fibrosis has been studied, suggesting its potential as a molecular marker in these conditions [23]. MiR-223 is predominantly expressed in myeloid cells and is involved in regulating innate immune responses, affecting the function of immune cells like neutrophils and monocytes and influencing inflammatory processes. Dysregulation of miR-223 has been observed in various inflammatory diseases, and its involvement in lung injury and fibrosis has been suggested [24]. Lastly, miR-342 is involved in immune response regulation and has been associated with inflammation, modulating the expression of genes involved in immune cell function and cytokine production, although its specific role in lung injury and fibrosis is not extensively studied [25].

In summary, miRNAs have become a focal point of research in understanding COVID-19. Based on existing literature, miR-21, miR-let-7, miR-146a, miR-146b, miR-155, miR-223, and miR-342, which are associated with inflammation, immune regulation, lung injury, and fibrosis, show promise in unraveling the complex mechanisms of COVID-19 and may serve as potential targets for diagnostics and therapeutics. Given the interplay between COVID-19 infection and these miRNAs, it is reasonable to expect that the expression patterns of these miRNAs during the acute phase of infection would differ from those observed during the mild or moderate phases. Therefore, analyzing the expression patterns of these miRNAs in samples obtained from individuals with different COVID-19 severity levels, in comparison to samples from healthy individuals, can provide valuable insights into the role of these miRNAs in the disease and its severity.

## 2. Materials and Methods

### 2.1. Subjects and Study Design

During a specific cross-sectional study conducted from January 2021 to June 2021 at Cairo’s Ahmad Maher Teaching Hospital in Egypt, a total of 272 individuals were enrolled as participants. This study aimed to collect various clinical samples, including nasopharyngeal, serum, and urine samples, from both infected individuals and healthy individuals. Ethical approval for the study was secured from the Ethics Committee of the General Organization of Teaching Hospitals and Institutes in Egypt, with reference number #HAM00125. The study adhered to the principles outlined in the Second Declaration of Helsinki, and all participants provided informed consent prior to their involvement. The individuals were divided into distinct groups: healthy donors (n = 57), individuals with mild COVID-19 cases (n = 106), individuals with moderate COVID-19 cases (n = 71), and individuals with severe COVID-19 cases (n = 39). The diagnosis of COVID-19 was confirmed through molecular testing of nasopharyngeal swabs. The study’s inclusion criteria mandated that participants be at least 18 years of age, while pregnant women and individuals with other respiratory viral infections were excluded from the study.

### 2.2. Definitions

The severity categorization of COVID-19 cases in Egypt was determined by the Ministry of Health, following the standards set by the World Health Organization (WHO) [26]. The WHO has established guidelines and criteria for classifying the severity of COVID-19 cases, taking into consideration various factors such as clinical symptoms, respiratory function, oxygen saturation levels, and imaging results. In summary, individuals who tested positive for SARS-CoV-2 but did not exhibit symptoms consistent with COVID-19 were categorized as having an asymptomatic infection. Those who displayed various signs and symptoms of COVID-19 (e.g., fever, cough, sore throat, headache, muscle pain, nausea, vomiting, diarrhea, loss of taste and smell) but did not experience shortness of breath, difficulty breathing, or abnormal chest imaging were classified as having mild COVID-19. Individuals who showed evidence of lower respiratory disease during clinical evaluation and maintained oxygen saturation (SpO2) levels of 94% on room air at sea level were classified as having moderate COVID-19. Those with an SpO2 level <94% on room air at sea level, a ratio of arterial partial pressure of oxygen to fraction of inspired oxygen (PaO_2_/FiO_2_) <30 breaths/min, or lung infiltrates exceeding 50% were categorized as having severe COVID-19. Cases of critical COVID-19 were identified in individuals who experienced respiratory failure, septic shock, and/or multiple organ dysfunctions. It is important to note that this study did not include individuals with critical COVID-19 upon hospital admission.

### 2.3. Data Collection and Sample Processing

Demographic information, clinical history, co-morbidities, and additional laboratory data were collected from medical records. The laboratory data included a comprehensive blood analysis, measurements of kidney functions, and markers of inflammation. Clinical samples were collected at the hospital on the same day of testing and placed in an RNA stabilization solution. These samples were then transported to the Virology lab at the Egyptian National Research Centre, Egypt. Urine and nasopharyngeal samples were centrifuged, and the resulting supernatants were stored at −80 °C. Whole blood was also centrifuged, and the serum obtained was stored at −80 °C.

### 2.4. Total RNA Extraction

Total RNA extraction from urine, nasopharyngeal, and serum specimens was performed using the QIAamp RNA Mini Kit (Qiagen, Hilden, Germany). The quantity and quality of the isolated RNA were determined using a NanoDrop spectrophotometer and stored at −80 °C.

### 2.5. SARS-CoV-2 Real-Time RT-PCR

A real-time RT-PCR assay for the quantitative detection of SARS-CoV-2 was performed according to the instructions of the Sansure COVID-19 Nucleic Acid Test Kit (Sansure Biotech Inc., Changsha, China).

### 2.6. MicroRNA Profile Expression

Reverse transcription was performed on 100 nanograms of RNA to convert it into first-strand cDNA. The miScript II RT Kit was used for this process. The expression levels of specific microRNAs such as has-miR-21 (477975_mir), has-miR-let-7 (4427975), hsa-miR-146a (4427975), hsa-miR-146b (4427975), hsa-miR-155 (4427975), hsa-miR-223 (4427975), and has-miR-342 (4427975) (ThermoFisher, Waltham, MA, USA) were analyzed in different phases of the disease and in various clinical types of patients with SARS-CoV-2 infection, as well as in healthy individuals. The miScript SYBR Green PCR Kit was used for the real-time PCR assay. The Ct values obtained for each miRNA were normalized using reference miRNA (miR-U6; 001093), and the relative levels in urine, nasopharyngeal samples, and plasma were determined. The amplification reactions consisted of an initial incubation at 95 °C for 5 min, followed by 40 cycles of denaturation at 95 °C for 5 s and annealing/extension at 60 °C for 30 s. All experiments were performed in triplicate. The amplification reactions were performed on a CFX Real-Time Polymerase Chain Reaction platform, and the fold change in expression was analyzed using the 2^^(−ΔΔCt)^ method.

### 2.7. Data Analysis

Statistical analysis was performed using the student’s *t*-test, one-way ANOVA tests for continuous and categorical variables. A significance level of *p*-value 0.05 was used. The spearman correlation matrix was constructed to analyze the correlation between microRNA expression and clinical data using R package. Receiver-operating characteristic (ROC) curve analysis was conducted to evaluate the sensitivity and specificity of each miRNA as potential biomarkers for COVID-19 using IBM SPSS Software v. 20.

## 3. Results

### 3.1. Demographic, Laboratory Markers and Clinical Symptoms

Table 1, Table 2 and Table 3 present a comprehensive overview of the demographic, biochemical, and clinical parameters of all the participants who took part in this study. The study included a total of 273 participants who were categorized into four distinct groups. Among these groups, the mild group consisted of 106 participants who received outpatient treatment without any specific complications, the moderate group included 71 participants who required hospitalization, the severe group comprised 39 participants who were admitted to the ICU due to specific symptoms, and there were 57 healthy participants in the control group. The mean age of the participants in each group was as follows: 36.7 ± 11.4 years (ranging from 25 to 49 years) for the healthy group, 38.5 ± 10.6 years (ranging from 28 to 55 years) for the mild group, 45.2 ± 9.3 years (ranging from 36 to 62 years) for the moderate group, and 61.8 ± 9.7 years (ranging from 48 to 71 years) for the severe group (refer to Table 1). Among the participants, the distribution of females within the healthy, mild, moderate, and severe groups was as follows: 14 (24.6%), 29 (27.4%), 32 (45.1%), and 13 (33.4%) (refer to Table 1).

Based on the findings presented in Table 2, it was observed that patients in the moderate and severe groups, who required hospitalization or were admitted to the ICU, had significantly higher serum levels of CRP (*p* < 0.001), D-Dimer (*p* < 0.001), Cr (*p* < 0.01), Urea (*p* < 0.01), and FBS (*p* < 0.01) compared to both the healthy group and the mild COVID-19 group. Moreover, when comparing the clinical characteristics of patients in the moderate and severe groups with those in the healthy and mild groups, it was found that fever (67.6% and 76.9%, respectively), cough (92.9% and 89.7%, respectively), and fatigue (87.4% and 94.8%, respectively) were the most commonly reported symptoms in both the moderate and severe groups. In addition, dyspnea (84.6%) and myalgia (77%) were more frequently reported by patients in the severe group compared to all the other groups. For a comprehensive overview of the clinical parameters of the subjects under study, including the healthy controls, please refer to Table 3.

### 3.2. Expression Analysis of Tested miRNAs

**Figure 1:**
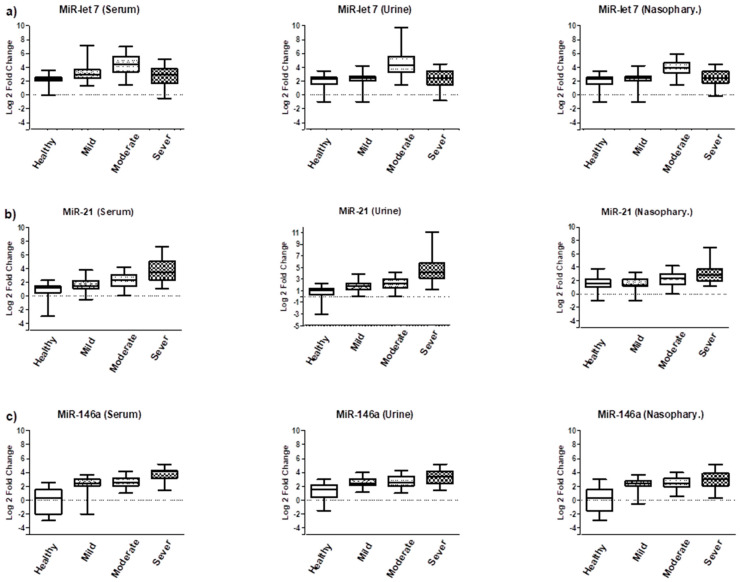
Comparison of miR-let-7, miR-21, and miR-146a expression levels in serum, urine, and nasopharyngeal samples of COVID -19 patients admitted to the ICU (Severe), hospitalized COVID-19 (Moderate), non-hospitalized COVID -19 (Mild), and control subjects (Healthy). (**a**) MiR-let-7 in serum, urine and nasopharyngeal samples, respectively; (**b**) MiR-21 in serum, urine and nasopharyngeal samples, respectively; (**c**) MiR-146a in serum, urine and nasopharyngeal samples, respectively.

**Figure 2:**
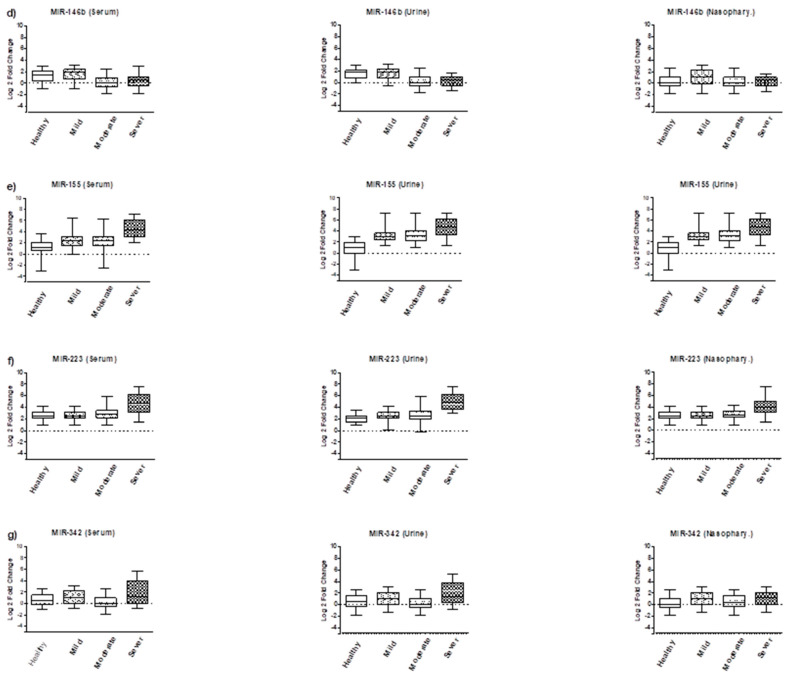
Comparison of miR-146b, miR-155, miR-223 and miR-342 expression levels in serum, urine, and nasopharyngeal samples of COVID-19 patients admitted to the ICU (Severe), hospitalized COVID-19 (Moderate), non-hospitalized COVID-19 (Mild), and control subjects (Healthy). (**d**) MiR-146b in serum, urine and nasopharyngeal samples, respectively; (**e**) MiR-155 in serum, urine and nasopharyngeal samples, respectively; (**f**) MiR-223 in serum, urine and nasopharyngeal samples, respectively; (**g**) MiR-342 in serum, urine and nasopharyngeal samples, respectively.

The findings of this study revealed that mild COVID-19 cases showed significantly higher levels of miR-21, miR-146a, and miR-155 in serum, urine, and nasopharyngeal samples compared to healthy individuals (*p* < 0.01), except for miR-21 expression in nasopharyngeal samples (*p* < 0.07). However, there were no significant differences in the expression levels of miR-146b, miR-let7, miR-223, and miR-342 between these groups. In moderate COVID-19 cases, the expression level of miR-let-7 was also upregulated (*p* < 0.01), in addition to the previously upregulated miR-155, miR-146a, and miR-21 observed in mild COVID-19 cases. Conversely, the expression level of miR-146b was downregulated (*p* < 0.05) compared to the healthy group. No significant differences were found in the expression levels of miR-223 and miR-342 between these groups. The most significant changes in miRNA expression profiles were observed in severe COVID-19 cases. The levels of miR-155, miR-146a, miR-21, and miR-223 were significantly upregulated (*p* < 0.001), while the expression level of miR-146b was downregulated (*p* < 0.01). Additionally, the expression level of miR-342 was dysregulated in all groups, as depicted in Figure 1 and Figure 2.

**Figure 3:**
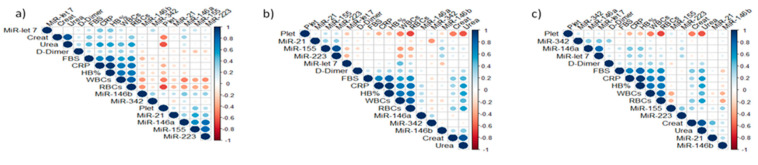

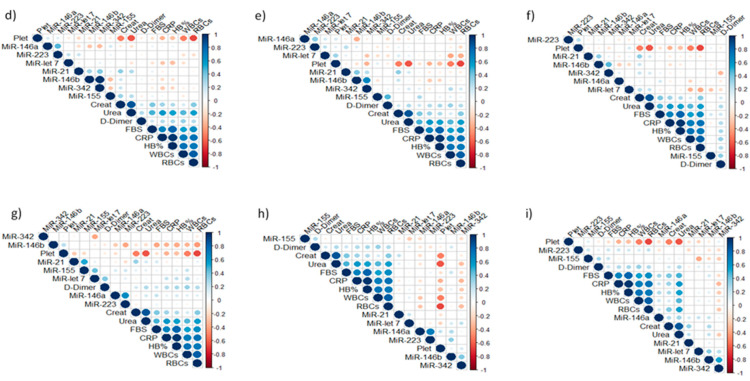
Spearman correlation matrix comparisons among the expression of miRNAs in serum, urine, and nasopharyngeal samples with the laboratory data of severe, moderate, and mild COVID-19 patients. (**a**) Serum of severe group; (**b**) Urine of severe group; (**c**) Nasopharyngeal samples of severe group; (**d**) Serum of moderate group; (**e**) Urine of moderate group; (**f**) Nasopharyngeal samples of moderate group; (**g**) Serum of mild group; (**h**) Urine of mild group; (**i**) Nasopharyngeal samples of mild group. Positive connections are shown in blue, while negative correlations are highlighted in red. The intensity of the color and the size of the circle are proportional to the correlation coefficients.

Based on the correlation analysis between miRNAs and laboratory markers presented in Figure 3, the severe COVID-19 group, which exhibited significantly upregulated expression levels of miR-21, miR-155, miR-let-7, miR-146a, and miR-223, showed significant positive correlations with platelets (*p* < 0.01), CRP (*p* < 0.001), HB% (*p* < 0.01), urea (*p* < 0.02), and D-Dimer (*p* < 0.05), and significant negative correlations with RBCs (*p* < 0.001) and Fasting Blood Sugar (FBS) (*p* < 0.05). In the moderate COVID-19 group, which had significantly upregulated expression levels of miR-21, miR-155, miR-let-7, and miR-146a, there was a significant positive correlation with creatinine (*p* < 0.03) and a significant negative correlation with RBCs (*p* < 0.001). In the mild COVID-19 group, which showed significantly upregulated expression levels of miR-21, miR-155, and miR-146a, there was a significant positive correlation with creatinine (*p* < 0.04) and D-Dimer (*p* < 0.02), and a significant negative correlation with CRP (*p* < 0.02), FBS (*p* < 0.01), HB% (*p* < 0.02), and RBCs (*p* < 0.001).

ROC curve analysis was performed for all miRNAs in different sample types, as shown in (Table 4, Table 5 and Table 6) and Supplementary Figure S4. The results of ROC analysis indicated that, in serum samples, miR-155 (Area under the Curve (AUC) = 0.927, *p* = 0.001) and miR-let-7 (AUC = 0.903, *p* = 0.017) can be used as biomarkers to distinguish severe COVID-19 patients from healthy individuals. In urine samples, miR-21 (AUC = 0.981, *p* = 0.001), miR-155 (AUC = 0.936, *p* = 0.001), and miR-let-7 (AUC = 0.893, *p* = 0.007) can serve as biomarkers for discrimination. However, no suitable candidate was found in nasopharyngeal samples to discriminate severe COVID-19 patients from healthy individuals. In serum samples, miR-155 (AUC = 0.942, *p* = 0.001), miR-let-7 (AUC = 0.903, *p* = 0.014), and miR-223 (AUC = 0.906, *p* = 0.004) can serve as biomarkers for moderate COVID-19 patients. However, only miR-let-7 (AUC = 0.873, *p* = 0.049) is effective as a biomarker in urine samples for the same group. In nasopharyngeal samples, miR-155 (AUC = 0.879, *p* = 0.002) demonstrates significant discriminatory ability between moderate COVID-19 patients and healthy controls. For mild COVID-19 patients, miR-155 (AUC = 0.929, *p* = 0.001), miR-let-7 (AUC = 0.972, *p* = 0.011), and miR-223 (AUC = 0.984, *p* = 0.001) can be utilized as biomarkers in serum samples. In urine samples, miR-146a (AUC = 0.929, *p* = 0.044), miR-155 (AUC = 0.919, *p* = 0.001), and miR-223 (AUC = 0.913, *p* = 0.001) are effective in distinguishing mild COVID-19 patients from healthy individuals. Lastly, in nasopharyngeal samples, only miR-146b (AUC = 0.962, *p* = 0.033) and miR-let-7 (AUC = 0.950, *p* = 0.035) can be employed as markers to differentiate between mild COVID-19 and healthy individuals.

## 4. Discussion

The primary objective of this study was to identify unique expression patterns of specific miRNAs (miR-21, miR-let-7, miR-146a, miR-146b, miR-155, miR-223, and miR-342) in COVID-19 patients with different disease states using various clinical samples, such as serum, urine, and nasopharyngeal samples, in comparison to healthy individuals. In recent years, circulating miRNAs have emerged as potential biomarkers for viral disorders and valuable tools for therapeutic applications, which could improve diagnostic accuracy [27,28,29,30,31]. A previous study suggested that measuring circulating miRNA-320a/b and D-dimer together enhances the diagnostic power for deep venous thrombosis. Differential expression of miRNAs has also been associated with the categorization of COVID-19 patients based on D-dimer levels. For example, another study found significant downregulation of miR-155, miR-146a, and miR-21, as well as a substantial over-expression of miR-342, in the circulating exosomes of COVID-19 patients with high D-Dimer levels [29,30]. Therefore, the selection of the panel of seven miRNAs in this study was based on their previous associations with inflammation, immune control, cell signaling, and viral infections [27,28,29,30].

Prior to the COVID-19 pandemic, miR-155 and miR-146a were identified as the first miRNAs stimulated by immune activation. They modulate the Toll-Like Receptor signaling pathway, which triggers the production of various inflammatory cytokines, type I interferon, and antiviral proteins [31]. A study by Soni et al. [32], reported higher levels of miR-155-5p in COVID-19 patients compared to healthy individuals. The study also investigated the effect of inhibiting miR-155 in the lungs of SARS-CoV-2-infected transgenic mice and found that the inhibition of miR-155 promoted survival, attenuated inflammation, and reduced the lung cytokine storm induced by the virus. In another study, the mean expression level of miR-155 was significantly higher in SARS-CoV-2-infected cell lines compared to control cell lines, suggesting that miR-155 plays a critical role in respiratory viral diseases by modulating antiviral responses, including inflammatory and immune responses [28].

The expression level of miR-146a exhibited a significant increase in A549 cells that were infected with the influenza virus. This upregulation of miR-146a played a role in promoting viral replication by inhibiting the production of interferon type I [33]. Moreover, it has been observed that elevating the expression level of miR-146a can mitigate lung cell damage by suppressing inflammatory responses [34]. Regarding miR-21, a study by Nersisyan et al. [35] demonstrated an eight-fold upregulation of miR-21-3p during lung infection caused by SARS-CoV-2. This upregulation facilitated the survival and replication of the virus. Another study investigating both miR-146 and miR-21 revealed that the levels of these miRNAs in the serum decreased in COVID-19 patients after treatment with the drug Tocilizumab [16]. Our findings are consistent with these previous studies and demonstrate that miR-146a, miR-155, and miR-21 were consistently upregulated in the serum, urine, and nasopharyngeal samples of COVID-19 patients with different disease severity groups (mild, moderate, and severe) compared to the healthy control group.

Regarding miR-let-7, a study by Pessôa et al. [36] demonstrated that its over expression led to a reduction in the expression of inflammatory cytokines and chemokines (IL-6, IL-8, and TNF-α). As for miR-223, alveolar epithelial type II cells are one of the main targets of SARS-CoV-2. In the context of lung injury, the migration of neutrophils to the site of inflammation worsens the inflammatory response and causes damage to the tissues. MiR-223 is released in microvesicles derived from neutrophils and is involved in lung damage. These microvesicles then transfer miR-223 to alveolar epithelial type II cells to contribute to lung protection [37]. Another study proposed that miR-223 plays a role in the post-transcriptional regulation of the NLRs-inflammasome pathway, and its excessive activation has been suggested to contribute to the progression of inflammation and the development of a cytokine storm in cases of acute COVID-19 [38]. Our research aligns with these previous studies and demonstrates that miR-let-7 consistently exhibits upregulation in the serum, urine, and nasopharyngeal samples of moderate and severe COVID-19 patients. On the other hand, miR-223 consistently shows upregulation, specifically in the serum, urine, and nasopharyngeal samples of severe COVID-19 patients.

The role of miR-146b in SARS-CoV-2 infection remains controversial, as contradictory results have been published. Some authors reported that it acts as a dominant negative regulator of the innate immune response by decreasing NK cell degranulation and the expression of downstream factors in the Toll-like receptor signaling pathway [28]. In this study, the downregulation of miR-146b was associated with an increased expression of pro-inflammatory cytokines, such as IL-6 and TNF-α, in severe COVID-19 patients [39]. However, other studies have reported conflicting results, showing upregulation of miR-146b in COVID-19 patients compared to healthy controls [40]. Further research is needed to elucidate the exact role of miR-146b in COVID-19 pathogenesis. In summary, the differential expression patterns of miRNAs in COVID-19 patients reflect their involvement in inflammatory and immune responses, as well as viral replication and survival. These miRNAs show potential as biomarkers for disease severity and could provide insights into the underlying mechanisms of COVID-19 pathogenesis. However, it is important to note that further validation studies are necessary to confirm these findings and establish the clinical utility of miRNAs as diagnostic or prognostic markers for COVID-19.

In the severe COVID-19 group, we observed significant upregulation of miR-21, miR-155, miR-let-7, miR-146a, and miR-223, as discussed earlier. These specific miRNAs exhibited significant positive correlations with platelets, C-reactive protein (CRP), hemoglobin (HB %), urea, creatinine, and D-dimer levels. Additionally, they showed significant negative correlations with red blood cells (RBCs) and fasting blood sugar (FBS) levels. In the moderate COVID-19 group, we found significant upregulation of miR-21, miR-155, miR-let-7, and miR-146a. These miRNAs displayed significant positive correlations with creatinine levels and significant negative correlations with RBCs. Within the mild COVID-19 group, we observed significant upregulation of miR-21, miR-155, and miR-146a. These miRNAs demonstrated significant positive correlations with creatinine and D-dimer levels, and significant negative correlations with CRP, FBS, HB%, and RBCs. These findings partially align with a previous study on COVID-19 patients, which identified a significant correlation between elevated expression levels of miR-155 and clinicopathological features such as increased white blood cell (WBC) and neutrophil counts, as well as a decreased lymphocyte count [40,41]. Another study also reported a significant correlation between creatinine, urea levels, and differentially expressed miRNAs, suggesting the involvement of these miRNAs in the regulation of kidney function. It is well known that COVID-19 can lead to kidney injury [42].

Furthermore, our ROC curve analysis demonstrated that miR-155, miR-let-7, and miR-223 exhibited high sensitivity and specificity, indicating their potential as biomarkers for distinguishing COVID-19 patients from healthy individuals. Adding miR-21 to these three miRNAs could be used as a biomarker for monitoring severe COVID-19 status, while adding miR-223 could be useful for monitoring moderate COVID-19, and adding miR-146 could be beneficial for monitoring mild COVID-19. In conclusion, this study was the first to report differentially expressed miRNA profiles, including miR-21, miR-155, miR-let-7b, miR-146a, miR-146b, miR-223, and miR-342, in various COVID-19 severity groups and a healthy control group. These findings contribute to our understanding of the role of miRNAs in COVID-19 and suggest their potential as biomarkers for COVID-19 monitoring.

## Figures and Tables

**Table 1:** Demographic parameters of the participants.

Groups	Age (Mean & SD)	Male n (%)	Female n (%)	Total n (%)
Healthy	36.7 ± 11.4	43 (75.4)	14 (24.6)	57 (100)
Mild	38.5 ± 10.6	77 (72.6)	29 (27.4)	106 (100)
Moderate	45.2 ± 9.3	39 (54.9)	32 (45.1)	71 (100)
Sever	61.8 ± 9.7	26 (66.6)	13 (33.4)	39 (100)

SD; Standard Deviation, n; number.

**Table 2:** Comparisons of the biochemical data of the participants among different groups.

**Test**	**Healthy** **Mean (Range)**	**Mild** **Mean (Range)**	**Moderate** **Mean (Range)**	**Severe** **Mean (Range)**
HB%, g/dL	13.4 (12.2–14.5)	13.2 (11.5–14.2)	12.8 (12–13.9)	11.6 (10.0–13.2)
WBCs, ×10^3^/μL	7.8 (5.9–9.5)	8.5 (6.3–10.8)	9.6 (7.1–12.7)	10.0 (8.6–14.9)
RBCs, ×10^3^/μL	4.7 (3.6–5.5)	4.2 (3.3–5.3)	4.0 (2.8–5.6)	3.9 (2.4–5.8)
PLT, ×10^3^/μL	223 (160–285)	191 (166–273)	168 (110–235)	173.4 (107–240)
FBS, mg/dL	85.6 (73–110)	93 (88–120)	139 (92–271)	207 (112–405)
B. urea, mg/dL	29.5 (22–45)	37 (27–45)	49 (30–84)	69 (33–123)
Creatinine, mg/dL	0.86 (0.5–1.1)	0.95 (0.5–1.1)	1.0 (0.7–1.4)	3.8 (1.1–7.5)
CRP, mg/mL	36 (12–96)	72 (18–108)	290 (60–560)	810 (400–1200)
D-dimer, ng/mL	210 (170–400)	225 (180–550)	366.2 (242–693)	939.5 (892–1340)

HB. Hemoglobin; WBCs. white blood cells; RBCs. red blood cells; Plt. platelets; CRP. C reactive protein.

**Table 3:** Comparisons of the clinical parameters of the participants among different groups.

Test	Healthy (n = 57)	Mild (n = 106)	Moderate (n = 71)	Severe (n = 39)
Fever; n, %	0 (0)	24 (22.6)	48 (67.6)	30 (76.9)
Cough	5 (8.7)	81 (76.4)	66 (92.9)	35 (89.7)
Dyspnea	0 (0)	15 (14.2)	40 (56.3)	33 (84.6)
Sore throat	3 (5.2)	19 (17.9)	65 (91.5)	27 (69.2)
Diarrhea	6 (10.5)	22 (20.7)	39 (54.9)	23 (58.9)
Fatigue	10 (17.5)	69 (65)	62 (87.4)	37 (94.8)
Abdominal pain	5 (8.7)	29 (27.3)	34 (47.8)	17 (44)
Myalgia	0 (0)	12 (11.3)	36 (50.7)	30 (77)
Headache	17 (29.8)	92 (86.8)	51 (71.8)	21 (54)

**Table 4:** ROC curve analysis of the expression of miRNAs in serum samples of various COVID-19 groups vs. control group.

Group	Target	AUC	*p* Value	Sensitivity	Specificity
Severe Group	MiR-21	0.838	0.006	100	85.7
	MiR-155	0.927	0.001	96.6	87.3
	MiR-146a	0.853	0.045	100	86.7
	MiR-146b	0.857	0.093	100	94.3
	MiR-let-7	0.903	0.017	100	94.4
	MiR-223	0.800	0.033	100	90.6
	MiR-342	0.731	0.028	91	100
Moderate Group	MiR-21	0.851	0.004	86.7	71.4
	MiR-155	0.942	0.001	100	75
	MiR-146a	0.843	0.051	97.1	86.7
	MiR-146b	0.614	0.591	100	97.2
	MiR-let-7	0.903	0.014	100	90.6
	MiR-223	0.906	0.004	100	87.5
	MiR-342	0.764	0.012	90.9	100
Mild Group	MiR-21	0.836	0.006	93.3	85.7
	MiR-155	0.929	0.001	86.6	75
	MiR-146a	0.775	0.119	97.1	100
	MiR-146b	0.757	0.227	100	97.3
	MiR-let-7	0.972	0.011	100	96.9
	MiR-223	0.984	0.001	100	87.5
	MiR-342	0.863	0.001	91	100

**Table 5:** ROC curve analysis of the expression of miRNAs in urine samples of various COVID-19 groups vs. control group.

Group	Target	AUC	*p* Value	Sensitivity	Specificity
Severe Group	MiR-21	0.981	0.001	100	81.2
	MiR-155	0.936	0.001	100	90.9
	MiR-146a	0.829	0.122	100	80
	MiR-146b	0.866	0.006	95	82.5
	MiR-let-7	0.893	0.007	100	88.9
	MiR-223	0.884	0.011	100	81.5
	MiR-342	0.861	0.001	95.5	100
Moderate Group	MiR-21	0.810	0.001	100	87.5
	MiR-155	0.800	0.002	100	95.5
	MiR-146a	0.821	0.131	100	97
	MiR-146b	0.678	0.065	100	94.2
	MiR-let-7	0.873	0.049	100	96.3
	MiR-223	0.825	0.004	100	92.9
	MiR-342	0.727	0.020	94.8	100
Mild Group	MiR-21	0.866	0.001	100	75
	MiR-155	0.855	0.009	100	90.9
	MiR-146a	0.929	0.044	100	91
	MiR-146b	0.819	0.001	100	94.1
	MiR-let-7	0.919	0.001	100	96.3
	MiR-223	0.913	0.001	100	96.4
	MiR-342	0.876	0.001	100	93.2

**Table 6:** ROC curve analysis of the expression of miRNAs in nasopharyngeal samples of various COVID-19 groups vs. control group.

Group	Target	AUC	*p* Value	Sensitivity	Specificity
Severe Group	MiR-21	0.779	0.19	100	94.3
	MiR-155	0862	0.003	100	71.5
	MiR-146a	0.869	0.002	100	82.4
	MiR-146b	0.766	0.100	100	80.6
	MiR-let-7	0.806	0.074	100	87.8
	MiR-223	0.569	0.574	100	96.7
	MiR-342	0.784	0.017	87.5	100
Moderate Group	MiR-21	0.886	0.07	100	91.4
	MiR-155	0.879	0.002	90	78.6
	MiR-146a	0.767	0.022	100	87.3
	MiR-146b	0.839	0.116	100	98
	MiR-let-7	0.800	0.095	100	88.9
	MiR-223	0.788	0.019	100	90
	MiR-342	0.897	0.001	100	96.4
Mild Group	MiR-21	0.921	0.08	100	97.1
	MiR-155	0.769	0.028	100	71.4
	MiR-146a	0.677	0.130	100	87.5
	MiR-146b	0.962	0.033	100	96.8
	MiR-let-7	0.950	0.035	100	96.3
	MiR-223	0.667	0.175	100	93.3
	MiR-342	0.784	0.015	87.5	100

## Data Availability

The data generated and/or examined during this study are not publicly available due to an ongoing research endeavor but are available upon reasonable request from the corresponding author.

## Supplementary Materials

The following supporting information can be downloaded at: https://www.mdpi.com/article/10.3390/biom13121681/s1, Figure S1: curve analysis using Serum, Urine and Nasopharyngeal miR-21, miR-146a, miR-146b, miR-155, let-7b, miR-223 and miR-342 for discriminating control, mild, moderate and severe COVID-19 patients.
