# Supplementary material for: A Comparative Analysis of MicroRNA Expression in Mild, Moderate, and Severe COVID-19: Insights from Urine, Serum, and Nasopharyngeal Samples
Source: Biomolecules. 2023 Nov 21;13(12):1681. doi: 10.3390/biom13121681 (PMC10742216; doi:10.3390/biom13121681)
Supplement: Supplementary file 1 [file biomolecules-13-01681-s001.zip › biomolecules-2687477-supplementary.pdf]

	Serum	Urine	Nasopharyngeal
miR- 21	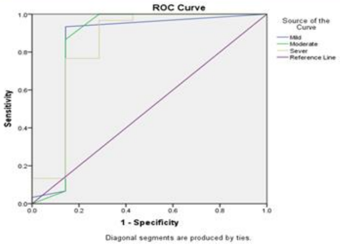	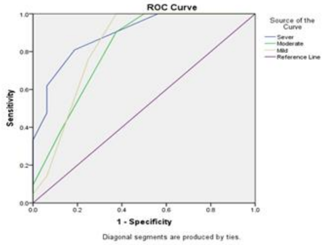	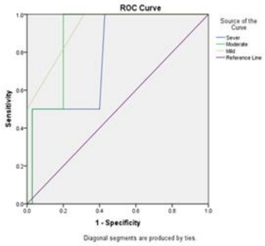
miR- let- 7	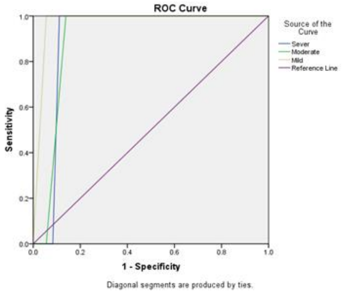	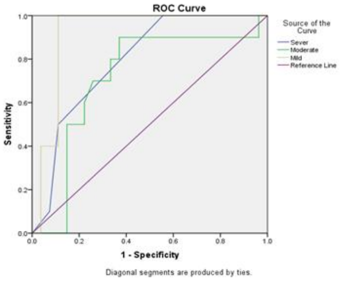	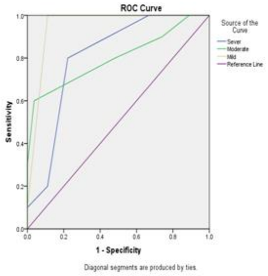

	Serum	Urine	Nasopharyngeal
miR- 146a	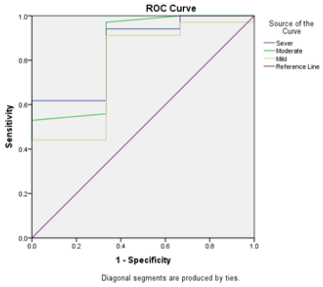	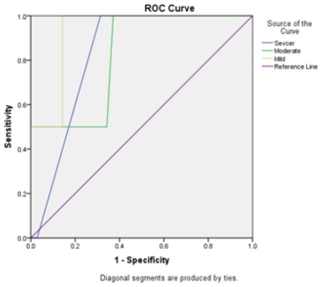	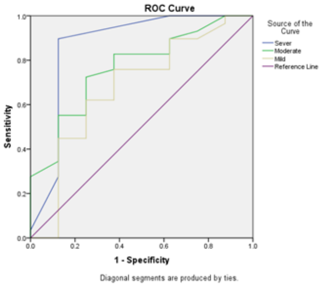
miR- 146b	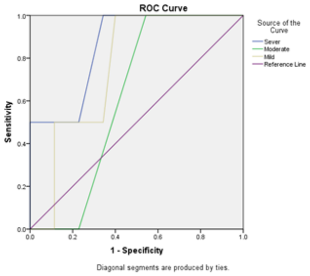	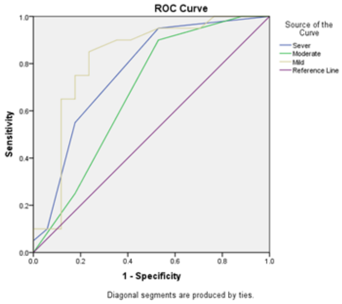	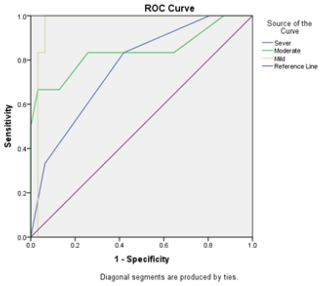

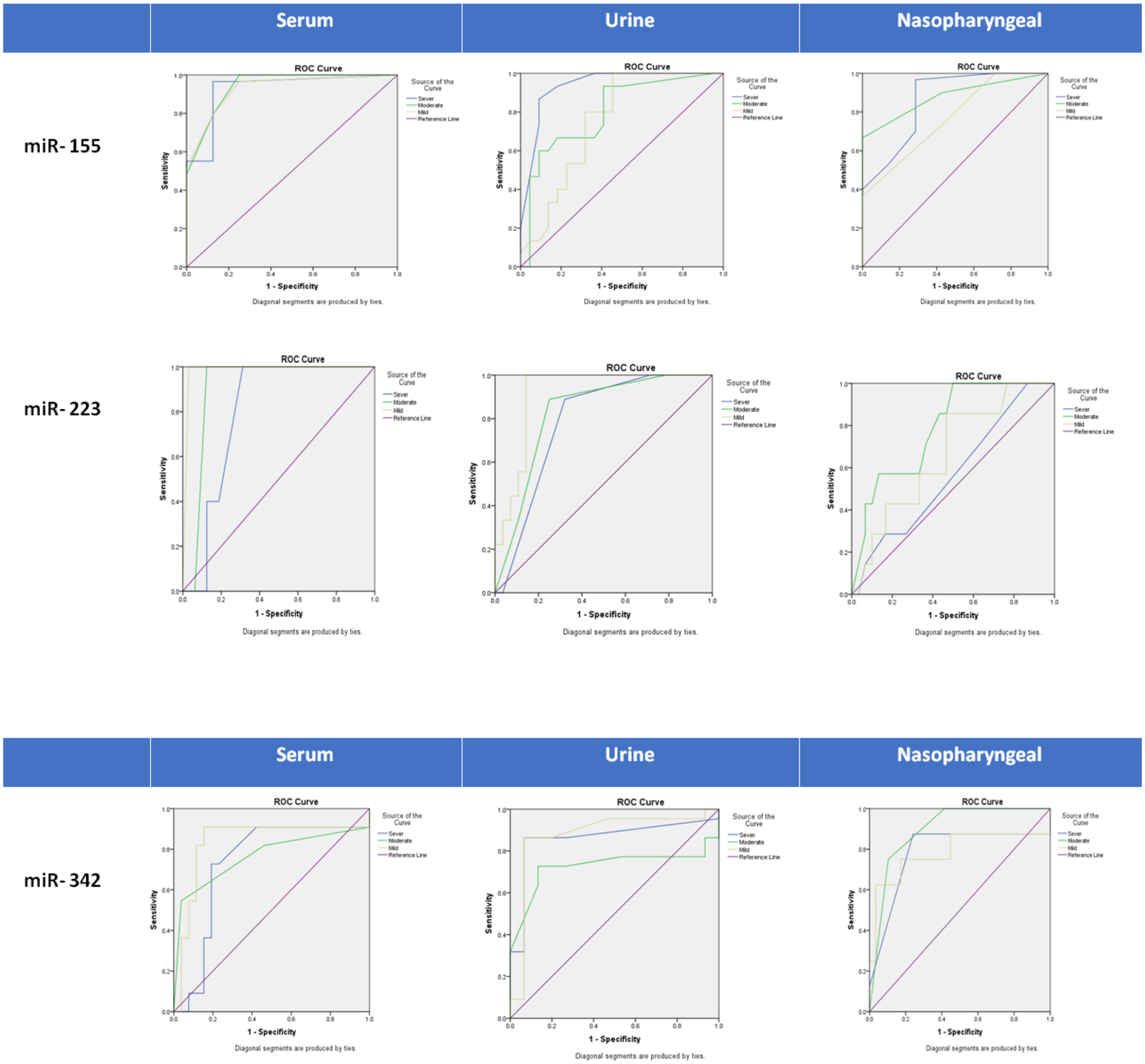

Figure S1. ROC curve analysis using Serum, Urine and Nasopharyngeal miR-21, miR-146a, miR-146b, miR-155, let-7b, miR-223 and miR-342 for discriminating control, mild, moderate and severe COVID-19 patients.
